# Neutrophil derived LTB4 induces macrophage aggregation in response to encapsulated *Streptococcus iniae* infection

**DOI:** 10.1371/journal.pone.0179574

**Published:** 2017-06-28

**Authors:** William J. B. Vincent, Elizabeth A. Harvie, John-Demian Sauer, Anna Huttenlocher

**Affiliations:** 1 Microbiology Doctoral Training Program, Department of Medical Microbiology and Immunology, University of Wisconsin-Madison; Madison, WI; United States of America; 2 Department of Medical Microbiology and Immunology, University of Wisconsin-Madison; Madison, WI; United States of America; 3 Department of Pediatrics, University of Wisconsin-Madison; Madison, WI; United States of America; Duke University, UNITED STATES

## Abstract

Immune cells sense and react to a multitude of factors including both host and microbe-derived signals. Understanding how cells translate these cues into particular cellular behaviors is a complex yet critical area of study. We have previously shown that both neutrophils and macrophages are important for controlling the fish pathogen *Streptococcus iniae*. Here, we report both host and bacterial determinants leading to the formation of organized macrophage aggregates as part of the host inflammatory response in a subset of infected larvae. Streptococcal capsule was a required signal for aggregate formation. Macrophage aggregation coincided with NFκB activity, and the formation of these aggregates is mediated by leukotriene B4 (LTB4) produced by neutrophils. Depletion, inhibition, or genetic deletion of leukotriene A4 hydrolase (Lta4h), which catalyzes the last step in LTB4 synthesis, resulted in the absence of macrophage aggregation. Larvae with impaired neutrophil function also had impaired macrophage aggregation; however, aggregate formation was partially rescued with the addition of exogenous LTB4. Neutrophil-specific expression of *lta4h* was sufficient to rescue macrophage aggregation in Lta4h-deficient larvae and increased host survival following infection. In summary, our findings highlight a novel innate immune response to infection in which specific bacterial products drive neutrophils that modulate macrophage behavior through eicosanoid signaling.

## Introduction

Immune cell populations communicate to carry out coordinated responses against a broad range of insults. For example, immune cell crosstalk via a positive feedback loop involving TNFα and IL17A between inflammatory monocytes and lymphocytes enhances the clearance of *Klebsiella pneumoniae* in a pulmonary infection model [1]. Moreover, neutrophils have been shown to either induce or suppress activation of the same immune cell populations [2–4]. Immune cell populations, including neutrophils and macrophages, also coordinate to carry out responses to sterile insults, for example during wound response [5,6]. Thus, immune crosstalk is important for modulating leukocyte responses in a diverse set of contexts.

Activated leukocytes release a variety of pro-inflammatory mediators to communicate with other cells, including the eicosanoid LTB4 [7,8], which is synthesized from leukotriene A4 by leukotriene A4 hydrolase (LTA4H). Leukotrienes play important roles in infection with bacteria [9–12], fungi [13], and parasites [14]. LTB4 enhances phagocytosis [9,15,16] and nitric oxide production [17] in macrophages, activates NADPH oxidase [18], and increases the production of antimicrobials [19,20]. LTB4 also stimulates the production of cytokines such as TNFα [12,21], IL-8 [22] and IL-6 [23] to further augment pro-inflammatory responses. Thus, LTB4 is a key mediator of the host responses to inflammatory stimuli.

Neutrophils are typically the first cells recruited to sites of bacterial infection or wounds [24]. While neutrophils were classically defined as simple effector cells of the innate immune system, understanding how neutrophils regulate immune behaviors is now an active field of study. It is now clear that in addition to their directly antimicrobial activities, activated neutrophils serve as modulators of the immune response by releasing pro-inflammatory molecules and cytokines/chemokines to recruit other immune cells to the infection site [25].

We used a zebrafish larval model and have characterized the formation of macrophage aggregates in response to infection with *Streptococcus iniae*. *S*. *iniae* is a significant fish pathogen in aquaculture, causing an estimated $100 million in annual costs worldwide, and can be an opportunistic pathogen in humans [26]. In a subset of infected fish, distinct macrophage aggregate structures form in the tail/trunk region, far away from the site of infection. Aggregate formation is specific, as these structures only form in presence of both live bacteria and bacterial capsule. Furthermore, we demonstrate that macrophage aggregation coincides with NFκB activation and that the presence of LTB4 signaling is required, as various means of disrupting LTB4 signaling all abrogate aggregate formation. Larvae with altered neutrophil function also have impaired formation of macrophage aggregates. Finally, neutrophil-specific expression of Lta4h is sufficient to rescue macrophage aggregate formation in Lta4h-deficient larvae and increases host survival.

## Results

### Macrophages are necessary for host defense to *S*. *iniae* infection

**Fig 1:**
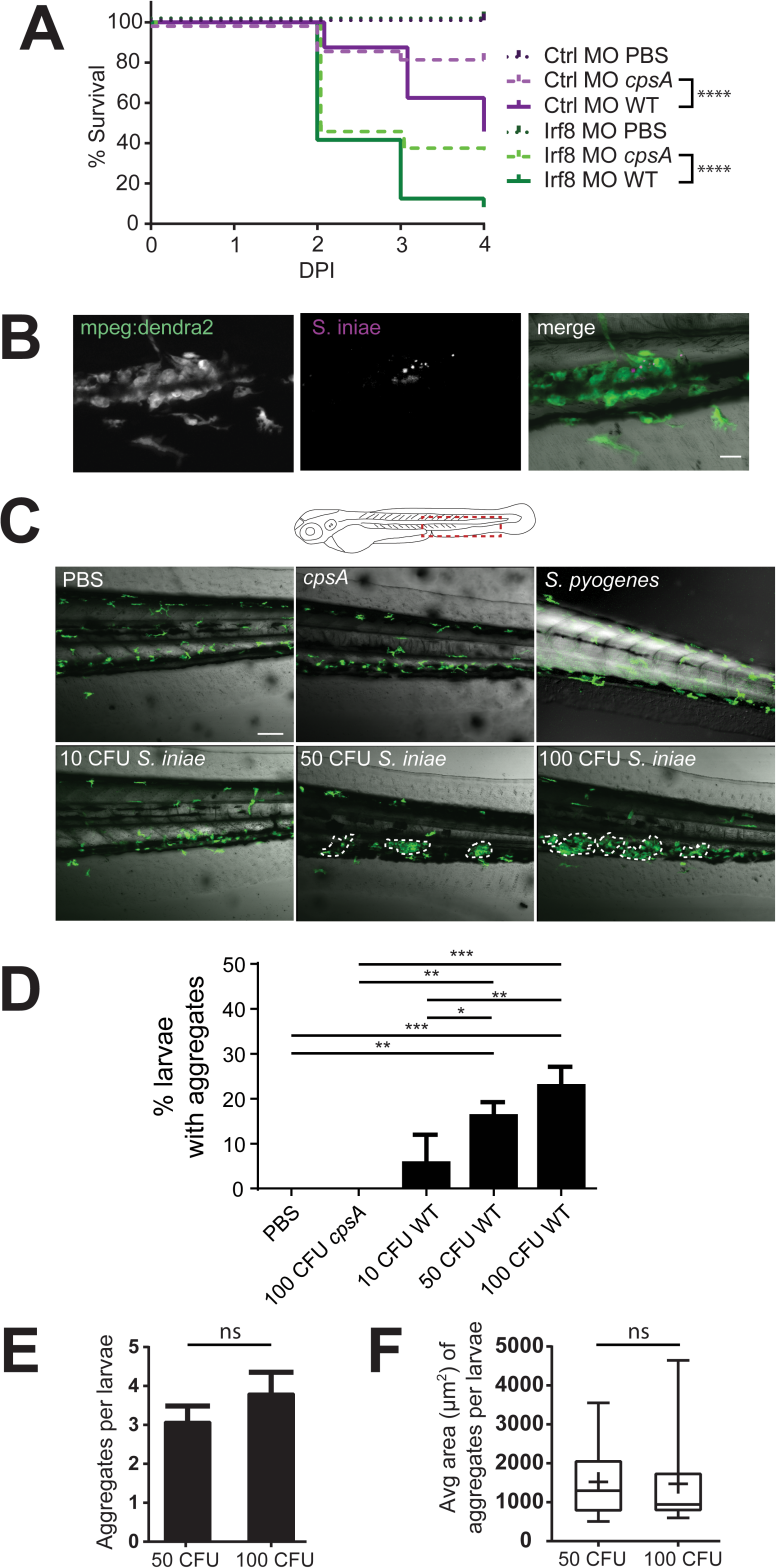
Macrophages are important for host defense and form aggregates in response to *S*. *iniae* infection. (A) Survival of *Tg(mpeg1*:*dendra2)* embryos injected at the single-cell stage with either the Irf8 (Irf8 MO) or standard control MO (Ctrl MO) following mock-infection with PBS or infection with 50 CFU *S*. *iniae*. Irf8 morphants infected with both WT and *cpsA S*. *iniae* have impaired survival (p < 0.0001), compared to control morphants. (B) *S*. *iniae* labelled with Cell Tracker Red are found within macrophage aggregates. Scale bar is 20μm. (C) *S*. *iniae* infection results in the development of macrophage aggregates in the trunk/tail of a proportion of infected fish by 24 hpi (diagram, red box). Representative 20X images of macrophage aggregates in *Tg(mpeg1*:*dendra2)* larvae 24 hpi following infection with PBS, *cpsA* mutant, 10 CFU, 50 CFU, and 100 CFU WT *S*. *iniae* as indicated. Scale bar is 80 μm. (D) Quantification of the average total percent of larvae forming macrophage aggregates from (C). (E) Average number of aggregates per larvae and (F) average aggregate size, as measured by the peripheral area of aggregates, at 24 hpi following infection with 50 or 100 CFU WT *S*. *iniae*. Area and number were not statistically significant across conditions. Data are from at least 3 independent experiments, with 24 larvae per condition.

We have previously shown that both neutrophils and macrophages are recruited to otic vesicle infection, and simultaneous depletion of neutrophils and macrophages increased susceptibility to infection with WT *S*. *iniae* and the avirulent, capsule-deficient *cpsA* mutant [27]. Additionally, we found that zebrafish with neutrophils harboring a neutrophil-specific dominant negative Rac2 D57N mutation, a model for leukocyte adhesion deficiency (LAD), have impaired neutrophil recruitment to localized sites of infection [28] and are also more susceptible to infection with WT *S*. *iniae* [27], but not capsule deficient *cpsA* insertion mutants. Since the specific role of macrophages in response to *S*. *iniae* infection has not been examined, we performed localized infection in the otic vesicle of larvae after transient depletion of macrophages using a morpholino targeting *irf8*. Irf8 morphants lack macrophages but have an increased number of neutrophils [29]. Macrophage deficiency markedly increased susceptibility to infection with 50 CFU *S*. *iniae* relative to control morphants (Fig 1A). Unlike Rac2 D57N larvae [27], Irf8 morphants also had increased susceptibility to *cpsA* infection (Fig 1A), supporting the importance of macrophages for controlling *S*. *iniae* infection and suggesting that neutrophils and macrophages perform non-redundant functions in host defense against *S*.*iniae*.

### Macrophages form aggregates in response to *S*. *iniae* infection

To examine macrophage behavior following infection with WT *S*. *iniae*, we infected the otic vesicle of *Tg(mpeg1*:*dendra2)* larvae that contain fluorescently labeled macrophages. Intriguingly, we observed the formation of distinct macrophage aggregates in a portion of infected larvae. *S*. *iniae* labeled with Cell Tracker Red dye could be found within macrophage aggregates (Fig 1B). While aggregates were present only in a minority of fish, they were distinct and were never observed in control larvae or after mock inoculations (Fig 1C and throughout). We defined these structures as an aggregation of 4 or more macrophages by 24 hours post infection (hpi) in the trunk region near the caudal hematopoietic tissue (CHT) (Fig 1C, diagram). Macrophages within aggregates were remarkably stable with limited motility compared to adjacent motile macrophages (S1 Movie). Macrophage aggregates were a specific response to *S*. *iniae*, as they were not observed following infection with the related pathogen *Streptococcus pyogenes* (Fig 1C). The proportion of larvae that developed aggregates increased in a dose-dependent manner (Fig 1C and 1D), but the average number of aggregates and aggregate size were not dependent on dose (Fig 1E and 1F). While larvae more reliably formed aggregates in response to 100 CFU *S*. *iniae*, this dose was rapidly lethal; thus to increase our ability to experimentally examine macrophage aggregation, we proceeded with a 50 CFU inoculum for all subsequent experiments.

### Streptococcal capsule is a determinant of macrophage aggregate formation

**Fig 2:**
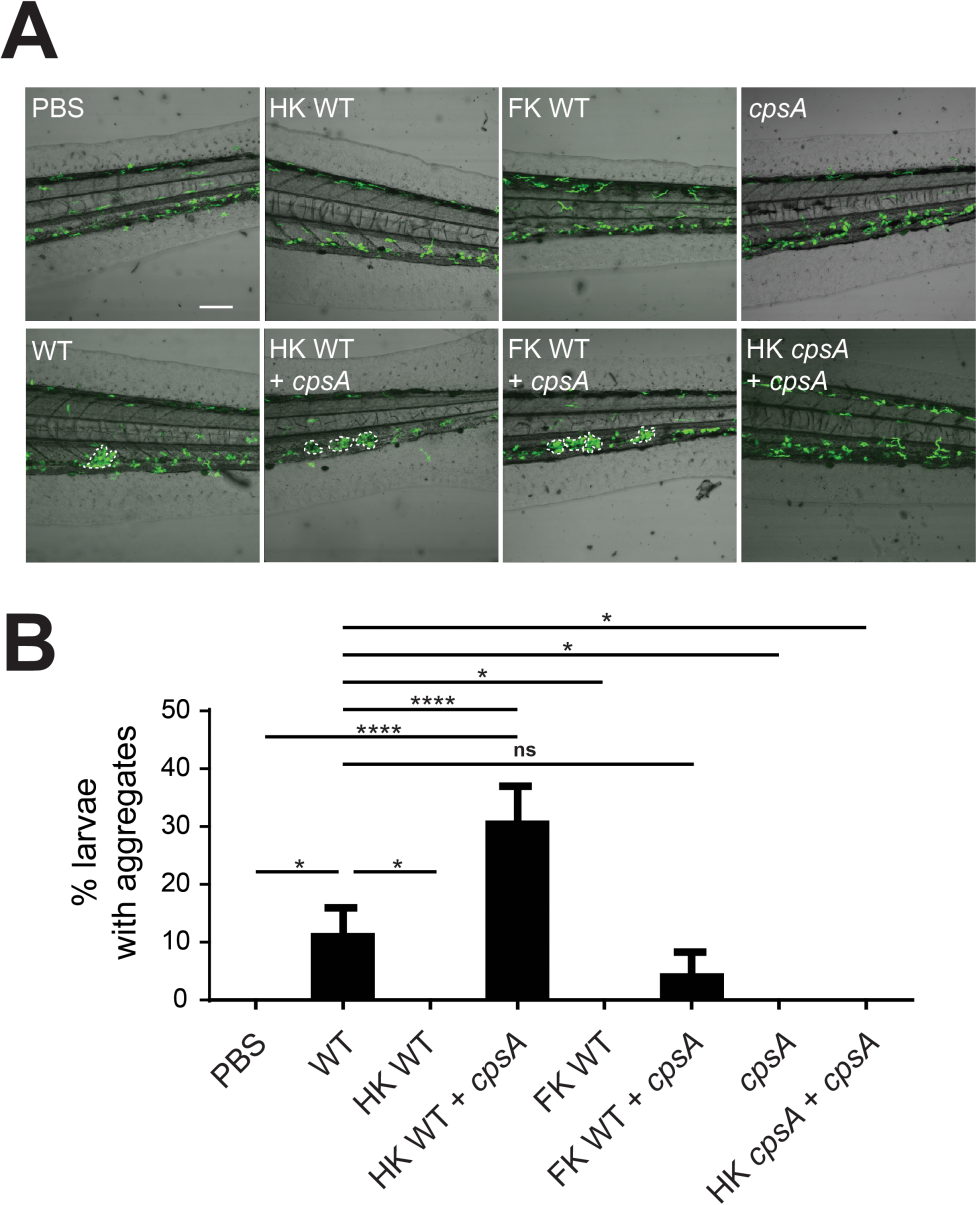
*S*. *iniae* capsule is a determinant of aggregate formation. (A) Representative 20X images of larvae at 24 hpi following inoculation with either PBS, 50 CFU WT *S*. *iniae* alone; 50 CFU equivalent heat killed (HK) or formalin killed (FK) plus 100 CFU *cpsA S*. *iniae*; 100 CFU *cpsA* alone; or 100 CFU live *cpsA* plus 100 CFU equivalent of HK *cpsA*. Scale bar is 80 μm. (B) Quantification of the average total percent of larvae forming macrophage aggregates from (A).

As aggregates only formed in response to *S*. *iniae* infection but not other bacterial infections, we asked if specific virulence determinants triggered this response. Given the critical role of capsule in *S*. *iniae* virulence, as well as the key role of macrophages in response to capsule deficient bacteria, we tested the hypothesis that aggregates may form in response to bacterial capsule. Macrophage aggregates did not form in response to *cpsA* infection (Figs 1C and 1D & 2A and 2B), even at infectious doses as high as 100 CFU. As in Fig 1, macrophage aggregates were induced in a subset of larvae infected with 50 CFU of WT *S*. *iniae* (Fig 2A and 2B). Strikingly, aggregate formation could be induced during *cpsA* infection by the addition of capsule *in trans*, using either heat-killed (HK) or formalin-killed (FK) WT *S*. *iniae*. Importantly, aggregates were not induced following the addition of HK *cpsA*, or HK or FK WT bacteria alone (Fig 2A and 2B). Taken together, our data suggest that initiation of macrophage aggregates requires the presence of both live bacteria and a specific pathogen determinant, the *S*. *iniae* capsule.

### Macrophage aggregates activate NFκB reporting and are dependent on LTB4 signaling

**Fig 3:**
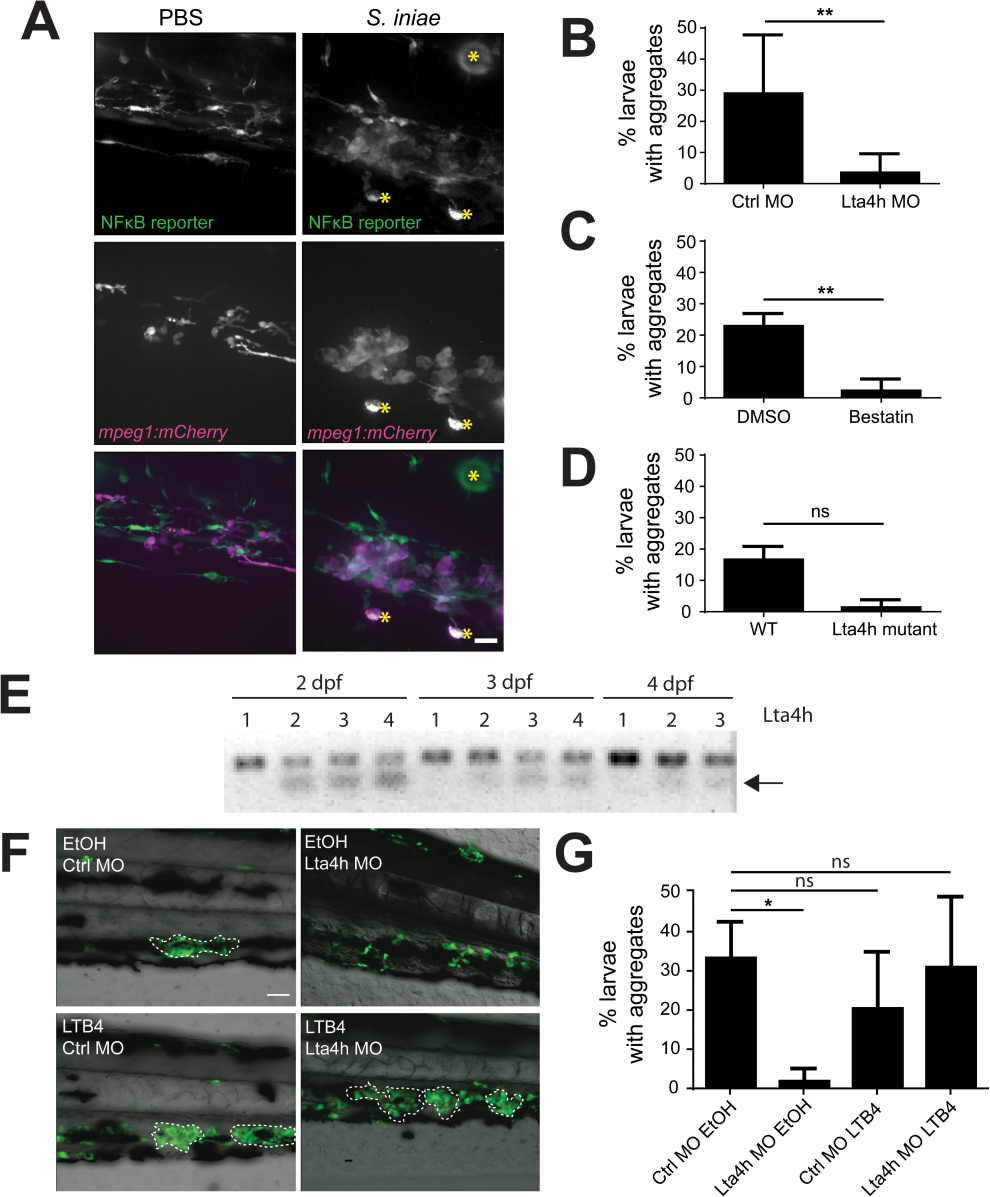
Disruption of Lta4h signaling abrogates macrophage aggregation. (A) NFκB reporter expression in *Tg(mpeg1*:*mCherry)* larvae at 24hpi following inoculation with PBS or 50 CFU *S*. *iniae*. Aggregate macrophages and adjacent cells show NFκB expression. Images at 40X; scale bar is 20 μm. Non-specific signal is indicated by yellow asterisks. (B and C) Average percentage of total larvae with macrophage aggregates at 24 hpi following inoculation with 50 CFU WT *S*. *iniae*. Aggregates fail to form in (B) Lta4h knockdown, p = 0.0087, average of 6 independent experiments; during (C) Lta4h inhibition with 100 μM Bestatin, p = 0.0022, average of 6 independent experiments; as opposed to relevant controls (Control MO, 0.1% DMSO, respectively), and were decreased in an (D) Lta4h mutant, p = 0.1000, average of 3 independent experiments. (E) RT-PCR of *lta4h* from mRNA extracted from 2–4 dpf zebrafish. The arrow denotes the presence of an alternative transcript in larvae injected with a splice-blocking Lta4h morpholino. Lane 1 = Ctrl MO, 2 = 100 μM Lta4h MO, 3 = 200 μM Lta4h MO, 4 = 500 μM Lta4h MO. (F) Representative 20X images of exogenous addition of LTB4 rescuing aggregate formation in Lta4h morphants. Larvae were treated with 30 nM LTB4 or 0.1% ethanol (EtOH). Scale bar is 80 μm. (G) Quantification of the average total percent of larvae forming macrophage aggregates from (F).

We next sought to determine if macrophage aggregates were associated with a change in inflammatory signaling. Using a zebrafish NFκB reporter line [30], we indeed found that macrophage aggregates co-localized with areas of increased NFκB reporter activity (Fig 3A). LTB4 can enhance NFκB signaling [31], and has been shown to play roles in maintaining immune cell structures during zebrafish infection [12]. Thus we examined if LTB4 signaling was an inflammatory signal that regulates *S*. *iniae* induced macrophage aggregates by targeting Lta4h, which catalyzes the final step in LTB4 synthesis. We found that inhibition of Lta4h activity by transient knockdown or chemical inhibition, or genetic mutation reduced macrophage aggregate formation following *S*. *iniae* infection (Fig 3B and 3C). Although the trend was clearly present, inhibition of macrophage aggregates fell just short of statistical significance in *Lta4h* mutant zebrafish (Fig 3D). These larvae did not have fluorescently tagged macrophages and thus were stained with L-plastin antibody which can also label neutrophils, and may have led to increased variance. Morpholino knockdown was efficient throughout the length of experiments (Fig 3E). Importantly, the absence of aggregate formation following Lta4h knockdown could be rescued following exogenous addition of LTB4 (Fig 3F and 3G). Of note, LTB4 did not induce aggregate formation in uninfected controls demonstrating that bacterially derived signals were still essential for this process. Taken together our data suggest that in addition to sensing live bacteria and the bacterial capsule, host signaling through LTB4 is required for the formation of *S*. *iniae-*induced macrophage aggregates.

### Neutrophil crosstalk drives macrophage aggregation

**Fig 4:**
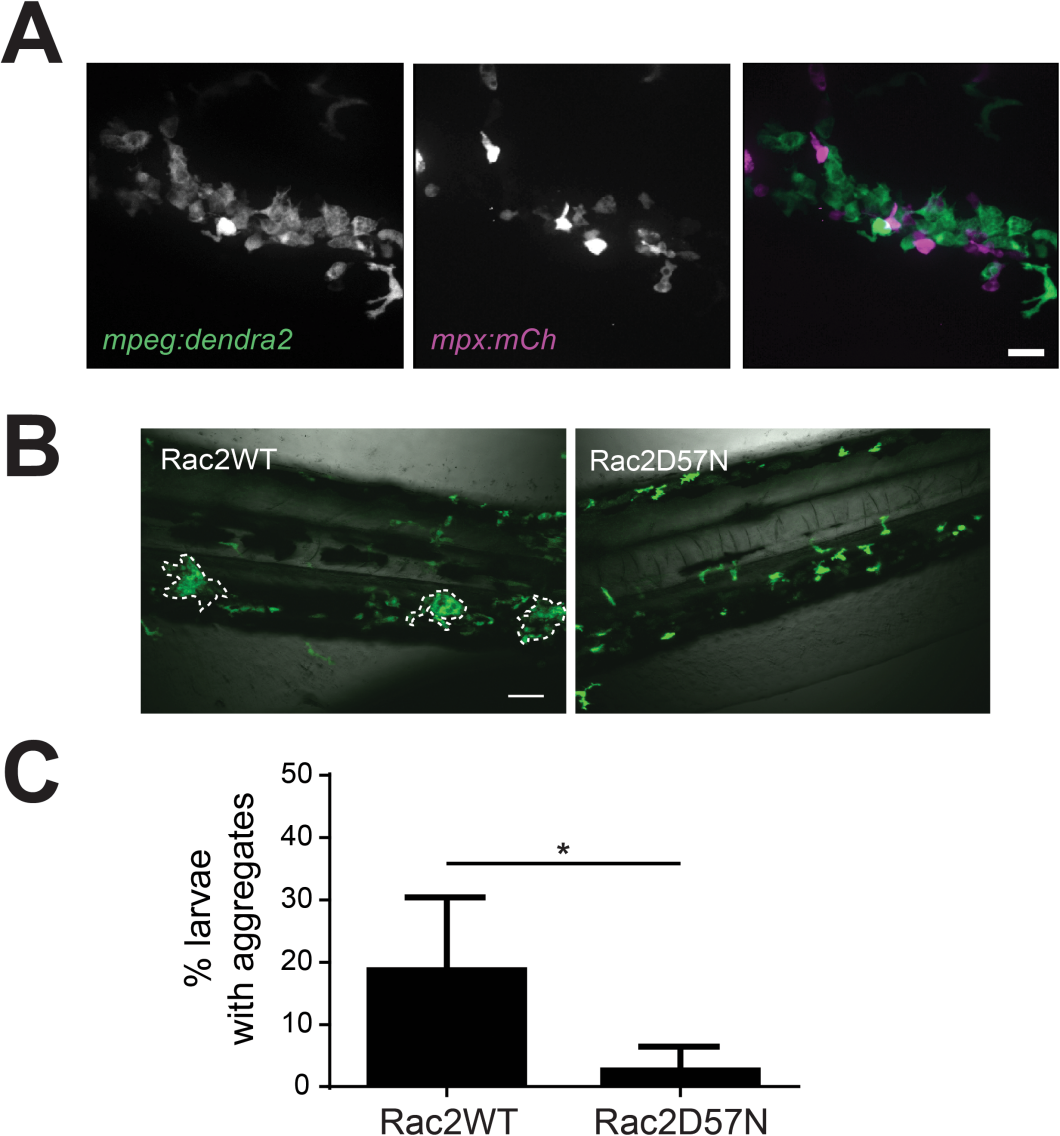
Neutrophil crosstalk drives macrophage aggregate formation. (A) Double transgenic *Tg(mpeg*:*dendra2)* x *Tg(mpx*:*mCherry)* larvae show that neutrophils are present in and around macrophage aggregates. Images at 40X; scale bar is 20 μm. (B) Representative 20X images of Rac2WT or Rac2D57N larvae at 24 hpi following inoculation. *Tg(mpx*:*mCherry-2a-rac2wt)* (Rac2WT) or *Tg(mpx*:*mCherry-2a-rac2d57n)* (Rac2D57N) were crossed to *Tg(mpeg1*:*dendra2)* and the resulting double transgenic larvae were infected with 50 CFU WT *S*. *iniae* or mock-infected with PBS. Rac2D57N larvae are defective for aggregate formation. Scale bar is 80 μm. (C) Quantification of the average total percent of larvae forming macrophage aggregates from (B).

Neutrophils are known to produce LTB4 and thus could be a potential source for the LTB4 that regulates macrophage aggregation. Consistent with this idea, we found that neutrophils regularly interacted with the periphery of macrophage aggregates (Fig 4A). To determine if neutrophils influence macrophage behavior, we examined macrophage aggregate formation in the absence of functional neutrophils. We used a zebrafish model of leukocyte adhesion deficiency in which neutrophils express a dominant negative Rac2 D57N mutation that results in impaired neutrophil recruitment to localized infection [27,28]. Surprisingly, Rac2 D57N-expressing larvae infected with 50 CFU *S*. *iniae* formed fewer aggregates than control larvae expressing Rac2WT (Fig 4B and 4C). Taken together, our data show that neutrophils normally interact with macrophages during *S*. *iniae* infection, and affect the formation of macrophage aggregates. When neutrophils were dysfunctional, the frequency of macrophage aggregation was lower, suggesting that in response to *S*. *iniae* infection, interactions between neutrophils and macrophages play a role in regulating the formation of macrophage aggregates.

### Neutrophil-specific expression of Lta4h rescues macrophage aggregation

**Fig 5:**
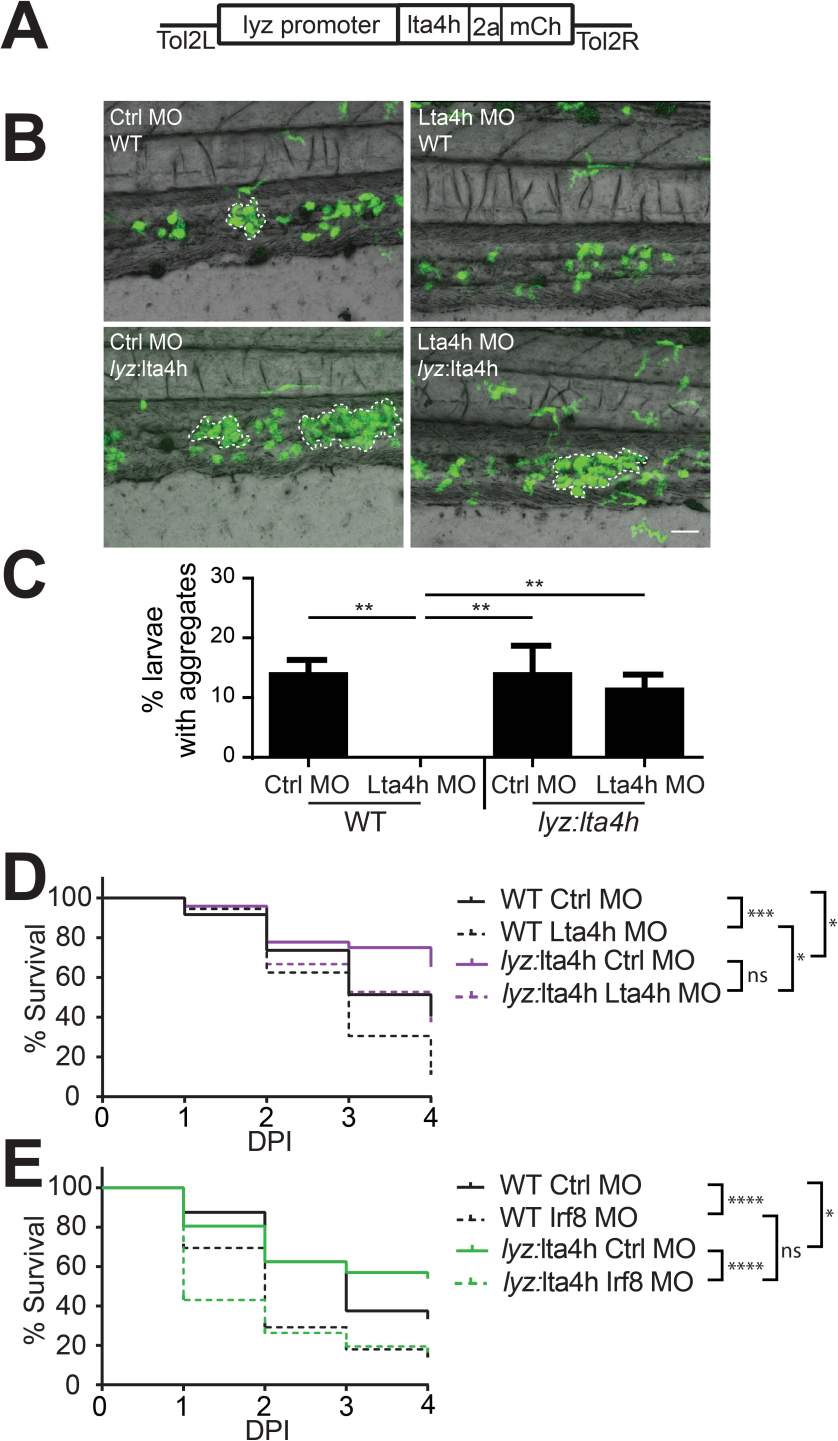
Neutrophil-specific expression of Lta4h rescues macrophage aggregation and correlates with host survival in Lta4h-deficient larvae. (A) Schematic of the *Tol2-lyz*:*lta4h-2a-mCherry* construct that was injected into AB-WT embryos to generate the *Tg(lyz*:*lta4h-2a-mCherry)* transgenic line. (B) The transgenic Lta4h sequence is non-targetable by morpholino knockdown. *Tg(lyz*:*lta4h-2a-mCherry)* (lyz:lta4h) or *Tg(mpx*:*mCherry)* (WT) was crossed to *Tg(mpeg1*:*dendra2)* and injected with either the Ctrl MO or Lta4h MO and monitored for macrophage aggregation at 24 hpi. Representative 20X images of WT or *lyz*:lta4h larvae at 24 hpi following inoculation with 50 CFU WT *S*. *iniae*, in both control and Lta4h morphant larvae. Scale bar is 80 μm. (C) Quantification of the average total percent of larvae forming macrophage aggregates from (B). (D) Survival of WT or *lyz*:*lta4h* larvae in either control or Lta4h morphant larvae infected with 50 CFU *S*. *iniae*. Compared with WT Ctrl MO larvae (black solid line), WT Lta4h MO larvae (black dotted line) have worse survival (p = 0.00437). Compared with *lyz*:*lta4h* Ctrl MO larvae (magenta solid line), *lyz*:*lta4h* Lta4h MO larvae (magenta dotted line) do not have significantly worse survival. However, *lyz*:*lta4h* Lta4h MO larvae (magenta dotted line) have significantly better survival than WT Lta4h MO larvae (black dotted line, p = 0.0142). (E) Increased survival in *lyz*:*lta4h* is macrophage dependent. Survival of WT or *lyz*:*lta4h* larvae in either control or Irf8 morphant larvae (lacking macrophages) infected with 50 CFU *S*. *iniae*. Compared with *lyz*:*lta4h* Ctrl MO larvae (green solid line), lyz:lta4h Irf8 MO larvae (green dotted line) have significantly worse survival (p < 0.0001). Compared with WT Irf8 MO larvae (black dotted line), *lyz*:*lta4h* Irf8 MO larvae do not have a significant difference in survival. Data are statistically pooled from at least 3 independent experiments, each with 24 larvae per condition.

Given the essential role of both LTB4 and neutrophils in the formation of S. iniae induced macrophage aggregates, we tested whether neutrophil-specific expression of *lta4h* was sufficient to rescue the macrophage aggregation defect in Lta4h deficient larvae. To test this hypothesis, we generated a transgenic line in which *lta4h* was expressed downstream of the neutrophil-specific promoter, *lyz* (Fig 5A), such that the *lta4h* transgene is not targetable by morpholino. Neutrophil-specific expression of *lta4h* was sufficient to rescue the defect in macrophage aggregation seen in Lta4h morphant larvae (Fig 5B and 5C). Neutrophils in *Tg(lyz*:*lta4h-2a-mCherry)* larvae also interacted with macrophage aggregates (S1A Fig), and neutrophil-specific expression of Lta4h did not affect macrophage aggregate size or number in *S*. *iniae*-infected control morphants (S1B and S1C Fig). Thus, our findings indicate that neutrophil-specific production of Lta4h is sufficient to rescue macrophage aggregation in response to *S*. *iniae* infection in Lta4h deficient larvae.

### Neutrophil-specific rescue of Lta4h correlates with increased host survival following *S*. *iniae* infection

Finally, we examined whether rescuing aggregate formation by neutrophil-specific expression of Lta4h affected survival following *S*. *iniae* infection. In a wild-type host background, Lta4h depletion increased susceptibility to *S*. *iniae* infection (Fig 5D, black dotted versus black solid lines). Lta4h depletion had no significant effect on survival in transgenic larvae that express Lta4h specifically in neutrophils *Tg(lyz*:*lta4h-2a-mCherry)* (Fig 5D, magenta dotted versus magenta solid lines). The *Tg(lyz*:*lta4h-2a-mCherry)* larvae had significantly increased survival after infection compared to WT larvae during Lta4h depletion (Fig 5D, magenta dotted versus black dotted line). Lta4h expression alone was sufficient to significantly improve survival in control morphants relative to wild type control morphants (Fig 5D, magenta solid versus black solid line). Importantly, these survival benefits were lost when macrophages were depleted using *irf8* morpholino (Fig 5E). Together, our data show that neutrophil production of Lta4h is sufficient to rescue both macrophage aggregation and host survival in Lta4h-deficient larvae.

## Discussion

Historically neutrophils were thought to function exclusively as effector cells of innate immune responses during infection, actively killing invading microbes. However, more recently their roles in contributing to inflammatory signaling and crosstalk with other immune cells has become more clear. Here, we have shown that neutrophil derived LTB4 modulates the macrophage inflammatory response to *S*. *iniae* infection in zebrafish larvae and that this response is dependent on the presence of both live bacteria and the production of capsule. These findings add to the immunomodulatory role of neutrophils during various coordinated innate immune responses and how they regulate other immune cells to affect host defense. Our results indicate that this regulation can correlate with relevant effects on the overall immune response to infection.

Both neutrophils [27] and macrophages (Fig 1A) contribute to controlling infection with wild-type *S*. *iniae*. However, in contrast to neutrophils, the absence of macrophages sensitizes larvae to infection with the avirulent *S*. *iniae cpsA* strain. This dichotomy led us to examine macrophage behavior and characterize the formation of distinct macrophage aggregates in infected larvae (Fig 1B–1E). Aggregates were specific to *S*. *iniae*, as aggregate formation is dependent on the presence of the *S*. *iniae* capsule (Fig 2), showing that the aggregative response is induced in response to specific pathogen derived signals. Aggregates were not observed during *S*. *pyogenes* infection (Fig 1C), highlighting the specificity of this response to the fish pathogen *S*. *iniae*. It would be intriguing to investigate if aggregate structures are found during streptococcal infections in a host species-specific manner, and if this host range has an impact on the presence or frequency of aggregates. The *cps* operon is conserved between *S*. *iniae* and other pathogenic streptococci such as *S*. *pneumoniae* and *S*. *agalactiae* [32–34], and our findings may have implications on host-specific responses after sensing streptococcal capsule during infection with other streptococcal species.

From the host perspective, maintenance of LTB4 signaling pathway is necessary to activate macrophage aggregation. LTB4 signaling has previously been shown to affect the size and stability of immune cell structures. *Mycobacterium marinum* infection in the zebrafish larvae leads to the formation of a complex granuloma of innate immune cells, and a lack of LTB4 signaling leads to earlier formation and larger granulomas [12,35]. In our model, the loss of Lta4h function instead abrogated the initiation of macrophage aggregates in response to *S*. *iniae* infection, as larvae lacking this pathway did not form aggregates (Fig 3B–3D). An interesting difference between the two models is that granulomas form throughout the larvae, whereas aggregates were only found in the region of the caudal hematopoietic tissue. LTB4 typically acts in a highly localized manner, however as evidenced by Fig 3G, this was not the case during *S*. *iniae* induced aggregate formation. The varying roles for LTB4 signaling in cell aggregation indicate the importance of this pathway during immune responses and cell interactions. It would be interesting to determine if exogenous LTB4 is sufficient to rescue aggregate formation during *cpsA* infection, to further discern the interplay of capsule sensing and LTB4 pruduction. Importantly, it is worth noting that we were unable to identify a signal able to induce aggregate formation in all larvae. Thus other bacterial determinants and/or host signaling must be necessary for successful macrophage aggregation.

We found that neutrophils interacted with macrophage aggregates (Fig 4A), and because they are known producers of LTB4, we examined the effect of limiting macrophage-neutrophil interactions on aggregate formation. Surprisingly, we found that when neutrophil crosstalk with macrophages is limited by expression of the Rac2D57N mutation in neutrophils, macrophage aggregate formation was impaired (Fig 4B and 4C), and neutrophil-specific Lta4h rescue was sufficient to restore aggregate formation in otherwise Lta4h-deficient larvae (Fig 5B and 5C). Exogenous LTB4 rescue suggests that in this system LTB4 is effective even when it is not at a localized source. Combined, these data suggest that early neutrophil-macrophage interactions and sensing of bacterial capsule at the site of inoculation induce LTB4 signaling, eventually leading to the formation of macrophage aggregates elsewhere.

A similar requirement for neutrophils in modulating the macrophage response to bacterial infection takes place in early granuloma formation in a mouse model of *M*. *tuberculosis* infection [36]. Additionally, neutrophils are recruited to macrophage-derived signals in the granuloma in the zebrafish *M*. *marinum* infection model [37]. Taken in this context, our data add to other studies that suggest neutrophils play key roles in modulating macrophage behavior. While we did not directly examine if aggregate forming larvae had lower bacterial burdens, rescuing aggregates with neutrophil-specific expression of Lta4h correlated with increased host survival following infection that was dependent on the presence of macrophages (Fig 5D and 5E), suggesting that manipulating neutrophil signaling can affect the outcome of infections through their effects on macrophages.

It has recently been shown that immune cell aggregation and granuloma formation can have striking parallels to tumor formation and maintenance [38,39]. Furthermore, it is now appreciated that neutrophils play critical roles both in promoting and inhibiting tumor formation and growth [40]. Our study now shows that neutrophil-macrophage interaction and LTB4 signaling are essential for the initiation of macrophage aggregation processes in response to signals from *S*. *iniae* during infection. Thus, it is tempting to postulate that further study of neutrophil crosstalk with other cell populations and related signaling pathways could lead to advances in our understanding of cell aggregation in the context of both infection and cancer biology.

## Experimental procedures

### Zebrafish maintenance and drug treatment

Zebrafish, embryos, larvae and adults were maintained in accordance and approval (protocol M005405) with the University of Wisconsin-Madison Research Animal Resources Center IACUC (Madison, WI, USA). For infections and live imaging, larvae were anesthetized in E3 medium containing 0.2 mg/ml tricaine (ethyl 3-amino-benzoate; Sigma-Aldrich). A light cycle of 10 h darkness and 14 h light was used. Wild-type AB fish were used to generate all transgenic lines, and the following transgenic lines were used in these studies: *Tg(mpx*:*mCherry)*, *Tg(mpeg1*:*dendra2)* [27], *Tg(mpx*:*mCherry-2A-rac2wt)* and *Tg(mpx*:*mCherry-2a-rac2d57n)* [28], *Tg(mpeg1*:*mCherry-histone2b)* [41] and *Tg(lyz*:*lta4h-2a-mCherry)* (this work, see below). Additionally, a previously described *lta4h*-deficient mutant with a retroviral insertion in the seventh exon of *lta4h* [12] was generously provided by Lalita Ramakrishnan. Embryos were obtained by natural spawning and were raised at 28.5°C in E3 medium as previously described. To prevent pigment formation, some larvae were maintained in E3 medium containing 0.2 mM *N-*phenylthiourea (Sigma-Aldrich, St. Louis, MO, USA). For infections and live imaging, larvae were anesthetized in E3 medium containing 0.2 mg/ml tricaine (ethyl 3-amino-benzoate; Sigma-Aldrich). Where indicated, E3 was supplemented with the following drugs immediately following infection and drug solutions were changed daily: 30 nM LTB4 (Cayman Chemical) and 0.1% ethanol, 100 μM Bestatin (Cayman Chemical) and 0.1% DMSO.

### Bacterial strains and microinjection of bacteria

*S*. *iniae* wild-type strain 9117 has been previously described [42,43]. *S*. *iniae* was prepared and microinjected into the otic vesicle of zebrafish aged 2–3 days post-fertilization (dpf) as described [27]. Where indicated, bacteria were labeled with 5 μM CellTracker Red CMPTX dye (C34552; Molecular Probes) according to the manufacturer’s instructions. Where indicated, heat-killing was achieved by placing a 50 CFU equivalent of WT *S*. *iniae* or 100 CFU equivalent of *cpsA* bacteria at 95°C for 30 min. Formalin-killing was achieved by resuspending in 1 ml of 4% paraformaldehyde and incubating at 37°C for 30 min.

### MO injection

All morpholino oligonucleotides (MOs) were purchased from Gene Tools, LLC (Philomath, OR, USA), resuspended in distilled water and stored at room temperature at a stock concentration of 1 mM. One-cell stage wild-type AB embryos were injected with 3 nl of morpholinos at the following concentrations: Irf8 MO, 400 μM; Lta4h (I7E8) MO, 500 μM. Comparable doses of the standard control MO were used in each experiment. The Irf8 [29], morpholinos were previously described. MO oligo sequences are as follows:

Lta4h: 5’- CAGTCTGATCAAGAGAAAGACTCGA-3’

Irf8: 5’- AATGTTTCGCTTACTTTGAAAATGG-3’

Elimination of macrophages in Irf8 morphants was confirmed by visual examination following injection into the *Tg(mpeg1*:*dendra2)* line that has fluorescent green macrophages. Confirmation of the Lta4h morpholino was achieved by RT-PCR using mRNA extracted from 2–4 dpf larvae.

Lta4h primers for RT-PCR:

lta4hF: 5’-TCTGAGAAGGAATATGTGGATGAA-3’

lta4hR: 5’-CAGCAAGAGATCTGTCTCCA-3’

### Generation of the transgenic *Tg(lyz*:*lta4h-2a-mCherry)* zebrafish line

DNA encoding *lta4h-2a-mCh* (zebrafish *lta4h* (Open Biosystems Clone ID 6961761, Accession CD760387, BC068394) was PCR amplified and inserted into a backbone vector containing minimal Tol2 elements for efficient integration, the *lyz* promoter for neutrophil-specific expression (Meijer et al., 2008) and an SV40 polyadenylation sequence (Clontech Laboratories, Inc., Mountain View, CA, USA). A viral 2A peptide linker sequence was used to facilitate production of multiple protein products from a single transgene [44]. One-cell stage wild-type AB embryos were injected with a 3 nl solution containing 25 ng/μl DNA and 35 ng/μl transposase mRNA and were grown at 28.5°C.

### Antibody staining

For examining aggregate formation in the *lta4h*-mutants, zebrafish were fixed in formaldehyde overnight at 4°C and immunolabeled as previously described [45] using rabbit antibodies to zebrafish L-plastin [46].

### Microscope analysis and live imaging

Anesthetized larvae were settled onto the bottom of a custom-made, glass-bottom dish. Fluorescence images were acquired with a laser scanning confocal microscope (FluoView FV1000; Olympus, Center Valley, PA, USA) using a numerical aperture 0.75/20X objective. Each fluorescent channel (488 nm and 543 nm) and differential interference contrast (DIC) images were acquired by sequential line scanning. Z-series were acquired using a 200–300 μm pinhole and 6–10 μm step sizes. Z-series were stacked using the FluoView FV1000 software. Fluorescence images acquired at 40x or 63X magnification were acquired using a spinning disk confocal microscope (Yokogawa CSU-X) with a confocal scanhead on a Zeiss AxioObserver Z.1 inverted microscope (NA 0.75/40x objective; NA 1.3/63X). A Photometrics Evolve EMCCD camera was used to acquire the images.

### Statistical analyses

Graphs displayed are the combination of averages over at least three independent experiments. Where single comparisons are displayed, a two-tailed t-test was performed. Where multiple comparisons are displayed, one-way ANOVA was performed. When comparing survival curves, data from at least three independent experimental replicates were pooled and analyzed using Cox proportional hazard regression analysis where the experimental conditions were included as group variables. The survival distributions were displayed in a graphical format using Kaplan-Meier plots. Significance throughout was defined as p < 0.05. Statistical analyses were performed using GraphPad Prism, version 6, and R statistical software, version 3.

## Supporting information

S1 Fig (A) Representative 63X images of macrophage aggregates in double transgenic *Tg(mpeg*:*dendra2)* x *Tg(lyz*:*lta4h-2a-mCherry)* larvae. Scale bar is 20 μm. (B) Average aggregate size, as measured by the peripheral area of aggregates, and (C) average number of aggregates per larvae at 24 hpi following infection with 50 CFU *S*. *iniae* in control or Lta4h morphants in a WT or *Tg(lyz*:*lta4h)* (*lyz*:lta4h) larvae background. Area and number were not statistically significant across all conditions.
(PDF)

S1 Movie Live-imaging time lapse of a macrophage aggregate in a *Tg(mpeg1*:*dendra)* x *Tg(mpeg1*:*mCherry-histone2b)* labeling the macrophage cytosol and nuclei, respectively. Non-aggregate macrophages (white arrows) nearby the aggregate are still motile, while macrophages within the aggregate are remarkably non-motile. A motile macrophage (red arrow) takes a circuitous path around the periphery of the aggregate.
(M4V)
