# Supplementary figures and images for: Neutrophil derived LTB4 induces macrophage aggregation in response to encapsulated Streptococcus iniae infection
Source: PLoS One. 2017 Jun 28;12(6):e0179574. doi: 10.1371/journal.pone.0179574 (PMC5489177; doi:10.1371/journal.pone.0179574)

**A**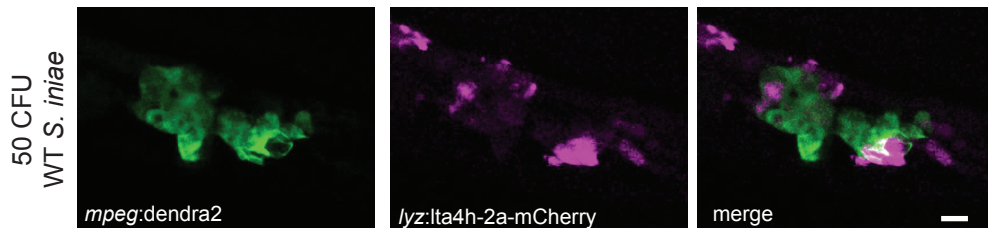**B**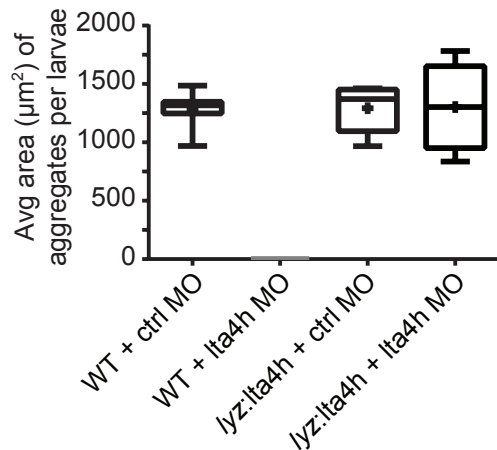**C**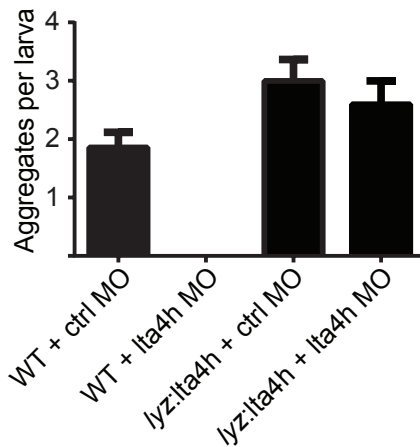

Supplement: S1 Fig — (A) Representative 63X images of macrophage aggregates in double transgenic Tg(mpeg:dendra2) x Tg(lyz:lta4h-2a-mCherry) larvae. Scale bar is 20 μm. (B) Average aggregate size, as measured by the peripheral area of aggregates, and (C) average number of aggregates per larvae at 24 hpi following infection with 50 CFU S. iniae in control or Lta4h morphants in a WT or Tg(lyz:lta4h) (lyz:lta4h) larvae background. Area and number were not statistically significant across all conditions. (PDF) [file pone.0179574.s001.pdf]
